# Supplementary material for: Content-rich biological network constructed by mining PubMed abstracts
Source: BMC Bioinformatics. 2004 Oct 8;5:147. doi: 10.1186/1471-2105-5-147 (PMC528731; doi:10.1186/1471-2105-5-147)
Supplement: Additional File 2 — The original results of the above study (non-essential files are deleted to keep the file size under the limit set by BMC bioinformatics). [file 1471-2105-5-147-S2.bz2 › chilibotAdditionalFile2/dip10/362ID8558064E5750/html/IL8_FY.html]

 


 **IL8** and **FY** 
  
Found 25 abstracts in PubMed, retrieved 10.  
 

 What does Google say? 
 PDF only 
| .edu only 

---

**Interactive relationship** (e.g. stimulation, inhibition, etc)

**Neutral relationship**- Results showed that supernatants from DARC  [ **FY** ]  ve concentrates contained higher levels of IL 8  [ **IL8** ]  up to days 14 15 of storage compared with DARC  [ **FY** ]  ve RCCs.  Ref: 10925048 Transfus Sci, 2000
- Augmented IL 8  [ **IL8** ]  would directly and or indirectly promote angiogenesis by binding to DARC  [ **FY** ]  and by inducing leukocyte infiltration and activation by binding to CXCR1 and CXCR2.  Ref: 9334359 J Exp Med, 1997
- The red blood cell Duffy antigen receptor for chemokines  [ **FY** ]  serves as a sink for the clearance of chemokines such as interleukin 8  [ **IL8** ]  IL 8  [ **IL8** ]  from the circulation.  Ref: 10964545 Clin Immunol, 2000
- Biopanning of phage peptide libraries with an anti Fy6 monoclonal antibody led to the identification of the motif Phe22 Glu23, the mutation of which altered the binding of both anti Fy6 and chemokines interleukin 8  [ **IL8** ] , MGSA, RANTES regulated on activation normal T cell expressed to DARC  [ **FY** ]  transfectants.  Ref: 9195930 J Biol Chem, 1997
- The Duffy Antigen Receptor for Chemokines  [ **FY** ]  DARC  [ **FY** ]  belongs to a family of erythrocyte chemokine receptors that bind C X C and C C chemokines such as interleukin 8  [ **IL8** ]  IL 8  [ **IL8** ] , monocyte chemoattractant protein 1 MCP 1 and regulated on activation, normal T cell expressed and secreted RANTES, but not macrophage inflammatory protein 1 alpha MIP 1 alpha or MIP 1 beta.  Ref: 9921412 Biomed Pharmacother, 1998
- Binding of IL 8  [ **IL8** ]  to both rheumatoid and non rheumatoid synovia was significantly reduced in the presence of anti DARC  [ **FY** ]  Fy3 and Fy6 monoclonal antibodies.  Ref: 12081195 J Pathol, 2002

**Non-interactive relationship** (e.g. studied together, co-existance, homology, etc.)

- We also determined serum tumor necrosis factor alpha TNF alpha, the phenotypes of the IL 8  [ **IL8** ]  binding Duffy blood group  [ **FY** ]  antigen receptor and the von Willebrand factor vWF plasma levels.  Ref: 12516654 Am J Reprod Immunol, 2002
- The lack of the chemokine binding was not likely to be due to the lack of glycosylation of the  **Fy**  GPA, since IL 8  [ **IL8** ]  was effectively bound to de N glycosylated erythrocytes.  Ref: 10873668 Biochem Biophys Res Commun, 2000
- mRNA for IL 8  [ **IL8** ]  binding chemokine receptors CXCR1, CXCR2, and the Duffy antigen receptor for chemokines  [ **FY** ]  DARC  [ **FY** ]  were found in all astrocytoma grades by reverse transcription PCR analysis.  Ref: 9334359 J Exp Med, 1997
- The involvement of DARC  [ **FY** ]  in chemokine binding was studied by incubating sections with blocking antibodies to DARC  [ **FY** ]  Fy3 and 6, to find out if these reduced 125I IL 8  [ **IL8** ]  binding.  Ref: 12081195 J Pathol, 2002
- The serum levels of IL 8  [ **IL8** ]  and monocyte chemotactic peptide MCP 1, two DARC  [ **FY** ]  binding chemokines, were investigated and related to this polymorphism.  Ref: 12739041 Rheumatol Int, 2003
